# Insulin Resistance: A Proinflammatory State Mediated by Lipid-Induced Signaling Dysfunction and Involved in Atherosclerotic Plaque Instability

**DOI:** 10.1155/2008/767623

**Published:** 2008-07-01

**Authors:** Fabrizio Montecucco, Sabine Steffens, François Mach

**Affiliations:** Division of Cardiology, Foundation for Medical Researches, University Hospital, 1211 Geneva, Switzerland

## Abstract

The dysregulation of the insulin-glucose axis represents the crucial event in insulin resistance syndrome. Insulin resistance increases atherogenesis and atherosclerotic plaque instability by inducing proinflammatory activities on vascular and immune cells. This condition characterizes several diseases, such as type 2 diabetes, impaired glucose tolerance (IGT), impaired fasting glucose (IFG), obesity, hypertension, dyslipidemia, and other endocrinopathies, but also cancer. Recent studies suggest that the pathophysiology of insulin resistance is closely related to interferences with insulin-mediated intracellular signaling on skeletal muscle cells, hepatocytes, and adipocytes. Strong evidence supports the role of free fatty acids (FFAs) in promoting insulin resistance. The FFA-induced activation of protein kinase C (PKC) delta, inhibitor kappaB kinase (IKK), or c-Jun N-terminal kinase (JNK) modulates insulin-triggered intracellular pathway (classically known as PI3-K-dependent). Therefore, reduction of FFA levels represents a selective target for modulating insulin resistance.

## 1. EPIDEMIOLOGY OF INSULIN RESISTANCE

Historically, the sweetness of urine and other body fluids in diabetic patients suggested that glucose had an important role in the physiopathology of this common disease. Thus, glucose metabolism and the insulin-glucose axis were the leading fields for scientific investigations. To emphasize this concept, diabetes was called “mellitus.” Not only hyperglycaemia is crucial for the diagnosis of diabetes and the development of clinical complications [1], but also increasing evidence demonstrated the involvement of insulin in the physiopathology of this disease. In fact, diabetes is a metabolic disease characterized by hyperglycaemia resulting from either defects in insulin secretion or insulin properties, or both. In the present review, we focus on defects of insulin properties, with particular regard to insulin resistance, which can be defined as a state of reduced responsiveness to normal circulating levels of insulin. This condition is a feature of various disorders, such as type 2 diabetes, which may range from predominantly insulin resistance with relative insulin deficiency to a predominatly secretory defect of insulin [1]. Insulin resistance is also implicated in impaired glucose tolerance (IGT) or impaired fasting glucose (IFG), both considered as “prediabetes” by the Expert Committee on the Diagnosis and Classification of Diabetes Mellitus [2–4], as well as in obesity, hypertension, dyslipidaemia (all disorders clustering in the so-called metabolic syndrome) [5], other endocrinopathies [6], but also in different diseases, such as cancer, infections or rheumatic, and autoimmune diseases [7–11]. Therefore, given the association between insulin resistance and different diseases, no epidemiological data are available, specifically focused on insulin resistance syndrome prevalence or incidence. Furthermore, only recently (in 1997) the WHO have accepted obesity as an epidemic public health burden in adults, without evaluating a well-defined method to monitoring the problem in children [12–15]. Finally, still few clinical studies on Asian and African cohorts focused on insulin resistance and related diseases have been published [16–19]. For all these reasons, to better define insulin resistance epidemiology syndrome requires more investigations. Mechanisms of insulin resistance remain also unknown. Insulin resistance was initially recognized as an “allergy” to insulin, with the production of antibodies anti-insulin [20, 21]. Further investigations showed that metabolism of both nonesterified fatty acids (NEFA) and free fatty acids (FFAs) was a crucial step in the development of insulin resistance [22, 23]. On the basis of these new evidences, Shafrir and Raz suggested in 2003 that diabetes should be now called “lipidus” instead of “mellitus” [24]. Given the importance of physiological effects of insulin during atherogenesis [25–27], there is a need to better clarify the complexity of mechanisms underlying insulin resistance.

## 2. MECHANISMS OF INSULIN RESISTANCE

Insulin is an anabolic essential hormone for the maintenance of glucose omeostasis, tissue growth, and development [28]. It is well known that insulin is secreted by the pancreatic *β* cells mainly in response to increased blood levels of glucose and aminoacids after the meals (extrinsic rhythm) [29]. In addition, the concentration of insulin in the blood displays regular variations independently from the food intake [30]. In fact, two rhythms with periods of 5–10 minutes and 60–120 minutes have been documented (intrinsic rhythm) [31–33]. The extrinsic rhythm was found altered in a lot of diseases including gestational diabetes [34], maturity onset diabetes of the young (MODY) 1 [35, 36], MODY 3 [37], and Chagas’ disease [38]. Furthermore, an altered plasma insulin secretory response has been also observed as an effect of aging processes [39]. On the contrary, the intrinsic rhythm has been found altered in various diseases, such as type 2 diabetes (i.e., MODY 2 as well as maternally inherited diabetes and deafness (MIDD)) [40, 41], obesity [42], and hypertension [43]. Several genetic and molecular studies have been performed to investigate the causes of the dysregulated plasma insulin pattern. Although genetic mutations account for a minor role in the large part of insulin resistance, an alteration of insulin signal transduction, which may be due to genetic mutations, could contribute to the impairment of insulin secretory profile and insulin resistance. Thus, mutations of glucokinase phosphorylated glucose, hepatic nuclear factor-4 alpha, hepatic nuclear factor-1 alpha, mitochondrial tRNALeu(UUR), and also insulin receptor genes have been found [35, 37, 44–46]. For instance, a mutation in the insulin receptor gene of the pancreatic *β* cells has been correlated with a defective insulin-mediated intracellular signal transduction [46]. On the other hand, obesity and increased FFA levels mediate insulin resistance by inducing a decreased IRS-1-associated phosphatidylinositol 3-kinase (PI3-K) activity [47]. In line with these findings, it has been shown that insulin resistance was reversed when obese persons lose weight [48]. However, this weight loss did not restore normal insulin pulsatiliy in Type 2 diabetes patients [49]. These data suggest that the defective insulin-mediated intracellular signal transduction is not the only cause responsible for insulin resistance, and that the molecular mechanisms of insulin resistance are not completely understood. We will discuss in the following the potential mechanisms contributing to insulin resistance which are currently under investigation.

### 2.1. Defects on insulin signaling

**Figure 1:**
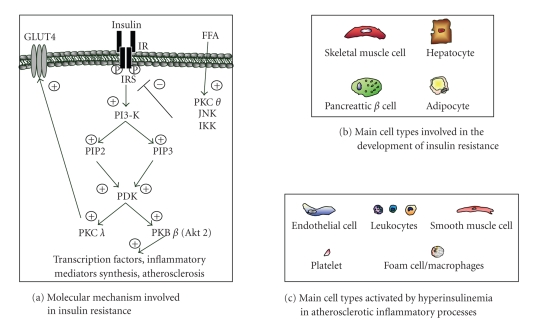
* Lipid signaling interference generates insulin resistance*. (a) Signaling through phosphatidylinositol 3-kinase (PI3-K) is crucial for insulin-mediated glucose transport in hepatocytes and skeletal muscle cells and for inflammatory protein and hormone secretion in adipocytes and pancreatic *β* cells. Free fatty acids (FFAs) induce a defective insulin-mediated signaling mainly through the activation of protein kinase C (PKC *θ*), inhibitor *κ*B kinase (IKK) and c-Jun N-terminal kinase (JNK). (b) Main cell types involved in the development of insulin resistance. (c) Main inflammatory cell populations involved in hyperinsulinemia-induced inflammatory states.

The insulin receptor is considered to play a critical role in insulin resistance. It is a member of the receptor tyrosine kinase family [50], composed of two *α*-subunits and two *β*-subunits linked together by disulphide bonds. Two isoforms of insulin receptors are known, exhibiting different affinity for insulin and distribution within tissues. Although it is tempting to suggest that differences in binding activity could contribute to insulin resistance, experimental evidence for the involvement of receptor isoforms or receptor hybrids remains controversial [29]. Apart from the insulin binding step to its receptor, the receptor intracellular tyrosine kinase domains (capable of intrinsic kinase activity) have been investigated in view of their possible implication in insulin resistance. A variety of scaffolding proteins, including insulin receptor substrate (IRS) proteins, casitas B lineage lymphoma (Cbl), or Cbl associated protein (CAP), bind to intracellular receptor sites and become phosphorylated [51–53]. IRS-1 and -2 are considered the most important proteins in regulation of glucose metabolism [54]. As shown in knockout mouse models, IRS-1 or IRS-2 inactivation causes insulin resistance [55, 56]. In addition, in vitro experiments showed an increased serine phosphorylation of IRS by tumor necrosis factor-alpha (TNF-*α*) or FFA stimulation, thereby causing impaired insulin signal transduction [54, 57]. Finally, a prolonged exposure to insulin (a typical condition in hyperinsulinemic patients) may result in a degradation of IRS protein [58]. All together, these data support IRS-1 and IRS-2 as crucial players in the development of insulin resistance. Furthermore, numerous studies aimed to identify downstream elements of IRS proteins in the insulin-mediated signal transduction pathway. As mentioned above, PI3-K is considered the central mediator [59]. Three different isoforms of this kinase have been identified: Ia, PI3-K/Akt, capable of generating phosphatidylinositol 4,5-bisphosphate (PIP2) and phosphatidylinositol 3,4,5-trisphosphate (PIP3); Ib, G protein regulated kinase; II, incapable of generating PIP2 and PIP3. As shown in Figure 1(a), activated PI3-K is responsible for the beginning of a complex phosphorylation cascade, involving the phospholipids PIP2, PIP3, the phosphoinositide-dependent kinase 1 (PDK1), the protein kinase B (PKB, also called Akt), as well as the protein kinase C (PKC). Akt mediates the effects of insulin on glucose transport (GLUT) [60], glycogen synthesis, protein synthesis, lipogenesis, and suppression of hepatic gluconeogenesis [59]. Once activated, Akt detaches from the plasma membrane and translocates into the nucleus through a still unknown mechanism [61], or activates different substrates, such as glycogen synthase kinase-3 (GSK-3) and transcription factors of the Foxo-family [59]. All these proteins and phospholipids are likely to be implicated in insulin resistance. For instance, a reduced PI3-K activity has been reported in skeletal muscle and adipocytes of patients with insulin resistance [62–64]. In addition, Akt activation has been found reduced in several diseases associated with insulin resistance [65, 66]. However, Akt involvement in insulin resistance is controversial, since other studies did not show any alteration of Akt activation in insulin resistance associated syndromes [67, 68]. On the other hand, insulin-induced PKC activation has been found altered in type 2 diabetic [69] or obese [70] patients. Therefore, although other studies are needed, all these observations suggest that a reduction of insulin-mediated intracellular signaling is crucial for the establishment of insulin resistance.

Another possible mechanism leading to insulin resistance might be an upregulation of protein-tyrosine phosphatases (PTPases), capable of functioning as negative regulators of the insulin-triggered pathway. Among various proteins, PTP 1B has been shown as a key regulator of insulin signaling [71–73]. Other phosphatases, such as ectonucleotide pyrophosphatase phosphodiesterase 1 (ENPP1, also known as PC-1), SH-2-containing inositol 5′-phosphatase 2 (SHIP2), and phosphatase and tensin homolog deleted on chromosome 10 (PTEN) have been shown to interfere with insulin sensitivity [74–76]. Although further investigations are warranted to very verify these hypotheses, these proteins may represent future potential targets for the treatment of insulin resistance.

### 2.2. Glucose metabolism

Glucose uptake into muscle and fat tissue depends on glucose transporter 4 (GLUT4) expression on the cell membrane. Insulin reduces glycaemia, mainly by inducing the secretion of this molecule by muscle and fat cells [77]. However, GLUT4 polymorphisms or mutations inactivating GLUT4 gene were not associated with insulin resistance [78]. In addition, GLUT4 concentrations in skeletal muscle of insulin resistant patients were not reduced [79]. This suggests that alterations in GLUT4 expression are not a primary cause for the development of insulin resistance. In this context, the correct functioning of insulin intracellular signalization appears essential. In fact, GLUT4 upregulation represents the final event of insulin signaling cascade [80–82]. Among various kinases (Figure 1(a)), Akt*β* has been shown to play an essential role. Recently, in vitro and in vivo studies suggested that PKB*β* alterations [83] or disruption [84] are responsible for the reduction of insulin-induced glucose uptake. Consistent with these data, recent studies in humans detected a missense mutation in the kinase domain of PKB*β* (Akt 2) associated with severe insulin resistance [85]. These data suggest that insulin signaling also plays crucial role in the regulation glucose homeostasis.

### 2.3. Inflammatory molecules

Recent evidence suggests that inflammation might be crucial for the development of insulin resistance [86]. Proinflammatory cytokines and acute-phase reactants are positively correlated with insulin resistance in metabolic syndrome patients [87]. Among these soluble mediators, interleukin (IL)-1 and IL-1 receptor antagonist (RA) have been implicated in the development of insulin resistance in humans [88, 89] and in rodents [90, 91]. IL-1RA is anaturally occurring cytokine and a member of the IL-1 family whose only function is to prevent a biologic response to IL-1 [92]. In humans, the blockade of IL-1 with IL-1 RA improves glycaemia and beta-cell secretory function and reduces markers of systemic inflammation [93]. Accordingly, IL-1 has been shown to induce insulin resistance mainly by inhibiting insulin-mediated signaling [94, 95]. Thus, IL-1 has to be considered as an important factor involved in insulin resistance. TNF-*α* and IL-6 are also of particular interest, because of their increased expression in adipose tissue and their capacity to induce insulin resistance [96]. Further evidence for the link between TNF-*α* and insulin resistance was provided by a study using blocking anti-TNF-*α* antibodies in obese rodents or TNF-*α* knockout obese mice [97]. In these animals, a reduced insulin resistance was obtained by the suppression of TNF-*α*. The possible molecular mechanism of TNF-*α*-induced insulin resistance may involve IRS-1 [98]. Surprisingly, the infusion of anti-TNF-*α* antibody in humans did not affect insulin resistance. Further investigations are needed to better understand these opposite results obtained in human and mice. On the other hand, the role of IL-6 in insulin resistance is also controversial. IL-6 interferes with the metabolism of both adipose and skeletal muscle tissues [99, 100], but has also a positive effect on skeletal muscle cell insulin sensitivity [101]. In addition, IL-15 has been shown to play a possible role in myocyte-adipocyte crosstalk, but only few studies are published at present to better clarify its role in insulin resistance [102]. Moreover, it is now established that hormones from adipose tissue hormones contribute to insulin resistance. For instance, leptin has been shown to reverse insulin resistance in mice with congenital lipodystrophy [103]. Administration of leptin to patients with lipodystrophy can increase the body fat content and reverse insulin resistance [104]. Resistin, a new adipocyte hormone [105], may be another important link between increased fat mass and insulin resistance [106]. Resistin decreases insulin-dependent glucose transport in vitro and increases fasting blood glucose concentrations and hepatic glucose production in vivo [106–109]. Similarly, the reduction of adiponectin could contribute to insulin resistance. Very recently, adiponectin has been showed as an anti-inflammatory and immunomodulatory molecule [110, 111]. In humans, adiponectin levels correlate with insulin sensitivity. Mice deficient in adiponectin are insulin resistant [112] and the administration of adiponectine to obese and insulin resistant mice has been shown to improve insulin sensitivity [113–115]. In addition, inflammatory mediators such as the proinflammatory chemokine monocyte chemotactic protein-1 (MCP-1) are believed to play a role in the pathogenesis of insulin resistance. Recent in vitro evidence suggests that MCP-1 induces insulin resistance in both adipocytes and skeletal muscle cells [116]. Finally, retinol-binding protein (RBP)-4 and tissue inhibitors of metalloproteinases (TIMP)-1 were recently described to contribute to insulin resistance in vivo, but the underlying mechanism remains unclear [117–119]. In conclusion, at the present state of knowledge, insulin resistance has to be defined as a complex syndrome involving not only glucose and lipids, but also several proinflammatory molecules.

### 2.4. Lipids and insulin resistance

Lipid abnormalities, such as increased circulating free fatty acids, are frequently associated with insulin resistance [120]. Lipid metabolism induces insulin resistance through a well-known cascade of events. The excessive fat intake causes an increased influx of triglycerides into the blood and an excess of plasma levels of FFAs, which induce insulin resistance, with consequent hyperglycaemia. The increased levels of glucose stimulate pancreatic *β* cells to secrete more and more insulin, generating hyperinsulinemia, which further triggers the elevation of triglycerides and closes the vicious circle [121]. When insulin secretion is not sufficient and elevated glucose levels prevail, diabetes becomes overt. Defective insulin secretion is a result of chronic exposure to elevated levels of fatty acids, which inhibits insulin gene expression by functioning as true toxic agents for pancreatic *β* cells [122]. “Lipotoxicity” depends on the interference with insulin-mediated intracellular signaling in various cell types (Figure 1). In particular, FFAs have been shown to activate PKC *θ* (Figure 1(a)), which not only interferes with insulin signaling (by inducing insulin resistance), but also is implicated in promoting proatherogenic mechanisms, such as endothelial dysfunction, growth, migration, and apoptosis of vascular smooth muscle cells, induction of adhesion molecules and oxidized low-density lipoprotein uptake of oxidized low-density lipoprotein by monocyte-derived macrophages [123]. Furthermore, it was recently shown that FFAs induce insulin resistance in muscle through the activation of inhibitor *κ*B kinase (IKK) and c-Jun N-terminal kinase (JNK) (Figure 1(a)) [124]. Therefore, FFAs induce insulin resistance in hepatocytes and skeletal muscle cells through the activation of different kinases (Figures 1(a) and 1(b)). Both FFAs from plasma and those released from stored triglycerides activate second messangers, which alter insulin signaling [125]. FFAs are also involved in modulating insulin production by pancreatic *β*-cells and cytokine secretion by hepatocytes, adipocyte, muscle cells, and inflammatory cells (Figure 1(c)). This strongly supports FFA as important proatherosclerotic agents.

## 3. ROLE OF INSULIN RESISTANCE IN ATHEROSCLEROTIC PLAQUE INSTABILITY

The development of atherosclerotic plaques is dependent on the interaction of multistep biochemical processes that lead to the plaque formation, maturation, and complication [126]. Plaque instability, rupture, and thrombosis are crucial events in the acute artery occlusion, which causes dramatic ischemic consequences in the heart, brain, and also peripheral tissues. Insulin resistance is considered to be a pivotal event in the increased risk of plaque instability through different pathways [127, 128]. High concentrations of insulin directly increase proinflammatory activities of leukocytes, which are involved in atherosclerotic plaque instability. In particular, insulin directly increases neutrophil and monocyte in vitro migration in response to chemokines secreted in atherosclerotic plaques [129, 130]. Insulin could also favor atherosclerotic plaque necrosis by accelerating macrophage death [131]. Furthermore, insulin induces in vivo production of matrix metalloproteinase-9 (MMP-9), which is responsible for plaque instability and rupture [132–134]. The pharmacologic or behavioral treatments to reduce insulin resistance have been shown to inhibit MMP-9 secretion [126, 135, 136]. On the other hand, insulin could also induce a serious atherothrombotic state, by increasing platelet resistance to antiaggregating agents [137] and production of procoagulatory factors, such as plasminogen activator inhibitor-1 (PAI-1), factor VII, factor XII, fibrinogen, and tissue plasminogen activator [126]. These evidences strongly support an emerging role of insulin in plaque instability and rupture. Further investigations are needed to better clarify the specific roles and the interactions of insulin and lipids on inflammatory cells.

## 4. CONCLUSION REMARKS

In the last years, the standard definition of insulin resistance has been shifted from a traditional “glucocentric” to a new “lipocentric” view [138]. Several soluble mediators are involved in the development of insulin resistance, through generating insulin signaling dysfunction. Among these, FFAs have to be considered as proatherosclerotic agents, capable of interfering with insulin signaling and provoking insulin resistance. New and selective therapies contrasting FFA effects may be promising targets for the treatment of insulin resistance. A possible promising strategy capable of reducing the consequences of excessive lipolysis and reorient FFA flux toward adipose tissue might be represented by peroxisome proliferator-activated receptor (PPAR)-*α* and -*γ* agonists [139]. PPAR-*γ* agonists have been recently shown to regulate trygliceride lipase in adipocytes in vitro and in vivo [140]. Furthermore, PPAR-*γ* has been shown as crucial in the control of differentiation of human monocytes in M2 macrophages, the subset of macrophages resident in atherosclerotic plaques with anti-inflammatory activity [141]. In vivo studies have also demonstrated that PPAR-*γ* agonists treatment in patients with type 2 diabetes mellitus is associated with a reduction in plasma NEFA levels [142–145]. However, two recent important clinical studies have shown an increase of acute cardiovascular outcomes induced by treatment with thiazolidinediones (PPAR-*γ* agonists) [146, 147]. On the other hand, although PPAR-*α* has been shown to have vascular and metabolic beneficial effects, the activity of PPAR-*α* agonists on lipid metabolism is still controversial [148, 149]. Therefore, further trials are needed to recommend the use of these pharmacological agents for reducing lipid-mediated insulin resistance.
